# Dermoscopy of Adnexal Tumors in Skin of Colour as a Diagnostic Challenge Extended to Dark Skin Tones

**DOI:** 10.7759/cureus.86501

**Published:** 2025-06-21

**Authors:** Emmanouil Karampinis, Nkechi Anne Enechukwu, Elizabeth Lazaridou, Biswanath Behera, Enzo Errichetti

**Affiliations:** 1 Dermatology, University of Larissa, Larissa, GRC; 2 Dermatology, Nnamdi Azikiwe University Teaching Hospital, Nnewi, NGA; 3 Dermatology and Venereology, Aristotle University of Thessaloniki School of Medicine, Thessaloniki, GRC; 4 Dermatology, Venereology and Leprology, All India Institute of Medical Sciences, Bhubaneswar, IND; 5 Dermatology, University of Udine, Udinese, ITA

**Keywords:** adnexal tumor, dermoscopy, pigmented bcc, skin of colour, trichoepithelioma

## Abstract

Skin adnexal tumors represent a wide spectrum of benign and malignant neoplasms, presenting morphological differentiation toward various adnexal structures. Skin of color variations in skin architecture and melanin production have been documented, possibly affecting the development and progression of those tumors, as well as their clinical and dermoscopy image. Therefore, we present a case series of clinical and dermoscopy presentations of adnexal neoplasms in skin of color individuals (Africans and Indians). We also compare these findings with 11 case reports and three case series that presented dermoscopy traits in adnexal tumors in dark-skinned patients found in medical literature.

The most common pigmentation trait found was brown blotches or brown structureless areas, corresponding to the increased epidermal pigmentation. Arborizing vessels, a characteristic of basal cell carcinoma (BCC), are frequently seen in trichoepitheliomas, cylindromas, and apocrine hydrocystomas, raising diagnostic challenges. Differentiating adnexal tumors from nodular BCC in skin of color poses a challenge due to overlapping features. Additional studies on the clinical evaluations of adnexal tumors are warranted. Early detection and prompt diagnosis are crucial, especially when differentiating benign adnexal tumors from malignant skin cancers, as this differential diagnosis significantly influences management and treatment outcomes.

## Introduction

The term skin of color (SoC) refers to individuals with melanin-rich skin, encompassing various racial identities such as Hispanic/Latino, Asian, African, Native American, Pacific Islander, and multiracial backgrounds. In dermatology research, there appears to be a relative lack of focus on SoC patients, as most studies have predominantly centered on white-skinned populations [1]. This gap is evident across multiple areas of dermatology, including macroscopic lesion descriptions and dermoscopic trait identification. For example, macroscopic images of inflammatory dermatoses in darker-skinned individuals may show clinical masking of erythema, or the erythema may appear dark red or violaceous in color, as seen in psoriasis, atopic dermatitis and tinea capitis [2].

Skin adnexal tumors comprise a broad and diverse group of both benign and malignant neoplasms, characterized by morphological differentiation towards various types of adnexal epithelium found in normal skin, including the pilosebaceous unit (follicular and sebaceous differentiation), eccrine, and apocrine structures [3]. Diagnosis of skin adnexal tumors is primarily based on histological evaluation, with classification typically determined by the predominant morphological component. Clinically and dermoscopically, these lesions are often difficult to assess, as they can closely resemble skin cancers. Many adnexal tumors, for instance, share similar dermoscopic features, making accurate differentiation a diagnostic challenge. Another important note is that adnexal tumors can serve as markers for syndromes linked to internal malignancies, such as trichilemmomas in Cowden disease and sebaceous tumors in Muir-Torre syndrome [3,4]. Their presence, particularly in large numbers, can further challenge the clinician, as identifying skin cancer among these lesions may become difficult.

Race-specific differences have been observed between skin structures and hair structure anatomy. African skin has larger keratinocytes, higher keratinocyte density, lower proteolytic activity, more fibroblasts, and slower skin shedding rates compared to Caucasian skin. Additionally, it features more cell layers in the stratum corneum compared to the thinner stratum corneum seen in individuals of Asian descent. Melanosomes in White skin are smaller and grouped in keratinocytes, are less dense and more numerous in the subcutaneous layer than in the basal layer. In contrast, Black skin has larger, individually dispersed melanosomes in keratinocytes, which are more numerous in the basal layer. Melanosomes are present in both the outer root sheath and the bulb of vellus hairs in Black individuals but absent in White individuals. Black hair contains more pigment and has larger melanin granules compared to hair from lighter-skinned and Asian individuals. Despite sharing some structural features across all races, certain differences have been noted, such as the fewer apocrine-eccrine mixed glands in White people compared to dark-skinned individuals [1].

Differences in skin structure, pigment production, and the origins of these structures may impact how adnexal tumors develop and progress in SoC, resulting in clinical and dermoscopic presentations that differ from those seen in the Caucasian population, affecting the differential diagnosis lists of those lesions as well. Therefore, we aim to present SoC and race-specific dermoscopy features of adnexal tumors and their respective mimickers and compare them with those characteristics proposed by reviews that are based on mixed populations.

## Case presentation

The following cases provide examination of the clinical presentations and dermoscopic characteristics of various adnexal neoplasms, offering insights into their distinct features and diagnostic considerations in SoC populations. Informed written consent was obtained from the patients for the open-access publication of this case series.

Case one

**Figure 1:**
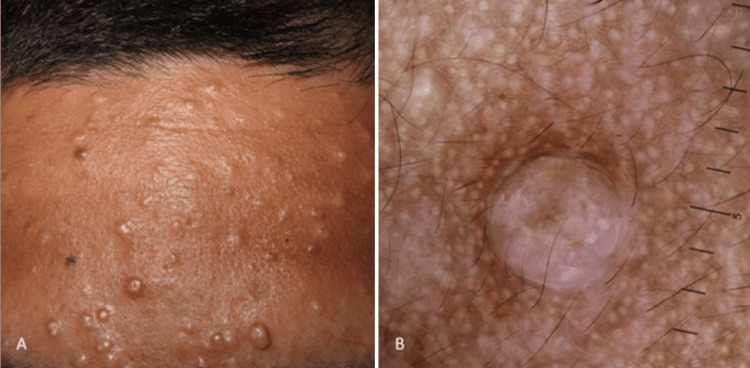
Trichoepitheliomas in SoC SoC: Skin of colour

A middle-aged Indian patient presented with multiple small, firm, skin-colored papules and nodules localized on the forehead. Dermoscopic examination revealed the presence of milia-like cysts along with subtle brown pigmentation (Figure 1). The clinical and dermoscopic features raised a differential diagnosis that included milia, sebaceous hyperplasia, syringomas, and trichoepitheliomas. Notably, the absence of crown or arborizing vessels and the specific localization of the grouped lesions to the forehead provided important diagnostic clues that narrowed the differential diagnosis list. Histopathological analysis confirmed the diagnosis of trichoepitheliomas.

Case two

**Figure 2:**
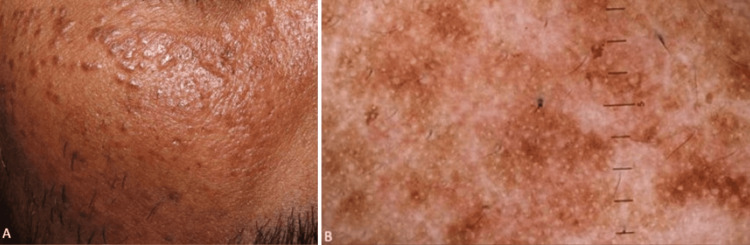
Syringomas in SoC SoC: Skin of colour

A 42-year-old Indian patient presented with multiple smooth-surfaced, yellowish to skin-colored or slightly pink papules clustered on the face, predominantly on the cheeks and periorbital region. Dermoscopic evaluation revealed brownish pigmentation interspersed with whitish structureless areas and numerous whitish dots on this background (Figure 2). These clinical and dermoscopic findings were consistent with a diagnosis of syringomas in a patient with SoC.

Case three

**Figure 3:**
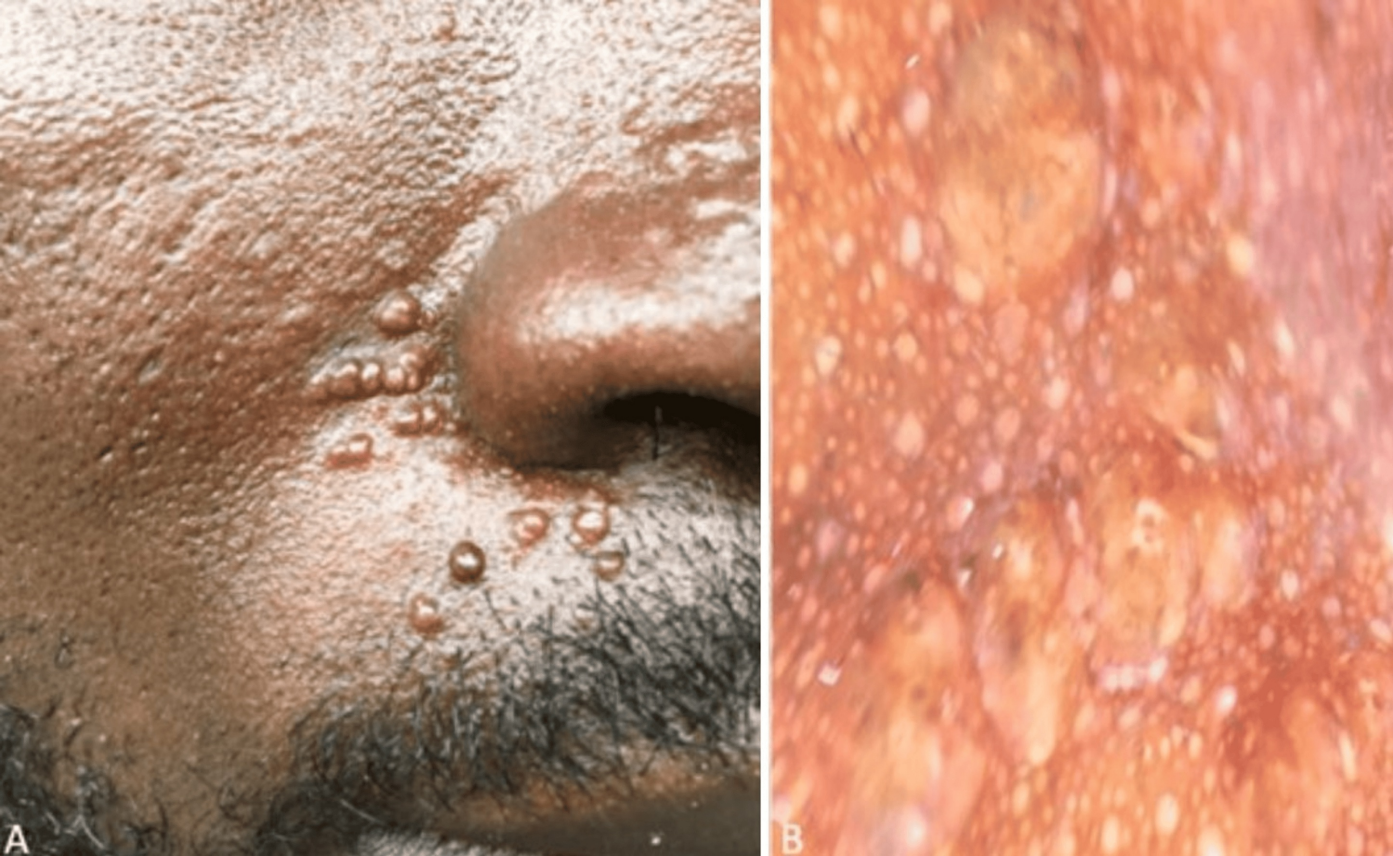
Trichoepitheliomas in SoC SoC: Skin of colour

A 38-year-old African patient presented with a four-year history of multiple firm, skin-colored to slightly translucent papules and nodules symmetrically distributed across the face. The lesions were asymptomatic and gradually progressed. Dermoscopic examination revealed white structureless areas localized to the papules, creating a noticeable contrast against the patient's dark skin (Figure 3). Based on the clinical presentation, dermoscopic features, and the histopathology assessment that followed, a diagnosis of trichoepitheliomas was established.

Case four

**Figure 4:**
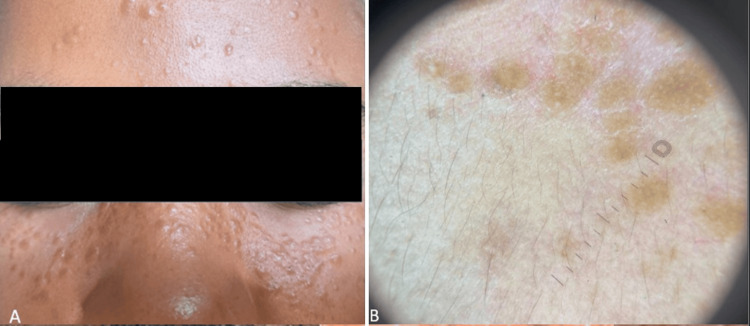
Syringomas in SoC

A young African woman presented with a three-year history of multiple small, skin-colored to slightly erythematous papules distributed over the forehead, bridge of the nose, and lower cheek areas. She reported a family history of similar lesions among close relatives. Dermoscopic examination revealed multiple scattered, rounded to oval structures ranging from yellowish-orange to brownish in color, along with a few milia-like cysts (Figure 4). The clinical distribution, chronicity, dermoscopic features, and positive family history were consistent with a diagnosis of syringomas in a patient with SoC.

Case five

**Figure 5:**
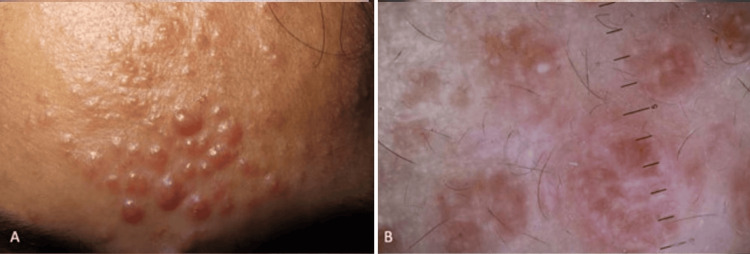
Cylindromas in SoC SoC: Skin of colour

A 36-year-old Indian woman presented with multiple rounded, dome-shaped papules localized on the forehead (Figure 5). The initial occurrence of those lesions was 15 years ago. Dermoscopic evaluation (Figure 5) revealed round to oval whitish structures suggestive of adnexal tumor lobules, set against a pink-white background. Based on the clinical appearance and dermoscopic features, a diagnosis of an adnexal tumor was considered. Histopathological examination confirmed the lesions to be cylindromas.

## Discussion

Adnexal tumors, as noted, pose a diagnostic challenge, and this difficulty extends to individuals with SoC. When a clinician examines a dark or skin-colored growth on a patient with SoC, more common skin conditions should initially be considered over adnexal tumors. Understanding the clinical features, location, and dermoscopic characteristics helps guide the dermatologist in their suspicion for these tumors.

Our study included 11 cases [5-15] featuring dermoscopy images of adnexal tumors in patients with SoC, along with three case series [16-18] that provided additional dermoscopic visuals that contained 28 dermoscopy examples (Table 1). Notably, most patients were of Indian descent; eight of the 11 case reports and two of the case series involved Indian patients. Most of the tumors were located on the face, except for poromas, which typically present on the lower limbs as well as on the palms and soles. Additionally, the clinical images revealed a diverse range of features that were largely nonspecific, highlighting the diagnostic challenges of these lesions.

**Table 1:** Cases with adnexal tumor dermoscopy in dark-skinned individuals focusing on pigment/brown structures, vessel pattern, white/yellow structures and surface traits

Lesions	Ethnicity	Location	Clinical presentation	Pigmented/Brown structures	Vessel description	White or yellow structures	Surface texture and keratin-related traits
Follicular Differentiation							
Trichoepithelioma [5]	Indian	Face	Firm, skin-coloured, dome-shaped papules	Not specific	Multiple arborizing or branching vessels	Rosettes over a whitish background	Milia-like cysts
Trichoepithelioma [6]	Indian	Face	Skin-coloured plaque	Not specific	Not specific	Not specific	Milia cysts in an erythematous background
Pilomatrixoma [7]	Indian	Cheek	Nodular lesion	Not specific	Erythema, linear irregular vessels and dotted vessels	Homogenous white area and white streaks	Not specific
Pilomatrixoma [7]	Indian	Ear lobe	Nodular lesion	Not specific	linear irregular vessels	Homogeneous white area and white streaks	Not specific
trichilemmal carcinoma [8]	Indian	Neck	Erythematous, hemorrhagic, and ulcerated nodules	Not specified	Multiple large and thin vessels	Yellowish-white structureless area	Ulceration
Sebaceous differentiation							
Sebaceous adenoma [9]	African-American	Trunk	Red-yellowish tumor of 1 cm	Not specific	Tortuous ramified vessels, more evident in the periphery of the lesion	Multiple pale yellow globules on an erythematous background	Crusts
Apocrine differentiation							
Papillary syringocystadenoma [10]	Dark-skinned Hispanic	Flank	Rose-colored papule	Not specific	Not specific	Yellowish-whitish structures separated by linear whitish structures on an erythematous background	Not specific
Eccrine differentiation							
Hidrocystoma [11]	Indian	Infraorbital area	Multiple small translucent cysts	Certain small cysts were bluish-colored	Not specific	Not specific	Not specific
chondroid syringoma [12]	Indian	Upper lip	A slow-growing, asymptomatic swelling	Irregular blotches with a few white lines	Erythematous rim at the periphery and linear curved vessels	White structureless area	Milia-like cyst
Poroma [13]	Indian	Foot	Pigmented nodule	Asymmetry, blue-green colour at the periphery, and multiple green-white ovoid nests	Polymorphic vascular structures in the centre	Not specific	Not specific
Cutaneous mucinous carcinoma [14]	Dark-skinned Taiwanese	Lower eyelid	Non-tender, slowly growing, erythematous nodule	Translucent gray globules encircled and focal brownish area	Linear irregular vessels located peripherally	Grayish-white areas in an arch-like pattern	Not specific
Cylindroma [15]	Indian	Face, scalp, and chest	Multiple nodules on the face, solitary nodule on the chest	Yellow-brown structureless areas and brown structures in the form of lines	Peripheral linear serpentine and branching vessels	White structureless areas	Not specific

Adnexal tumors in skin of color exhibit distinct dermoscopic patterns, particularly in pigmented/ brown structures, vascularity, white/yellow features, and surface texture. Melanin-related findings are commonly seen in trichoepitheliomas, melanotrichoblastoma, trichoepithliomas, poromas, cylindromas, and syringomas, appearing in most cases with brown structureless areas [5,6,12,18]. Fine brown peppering or pseudonetworks were also noted. Fine brown peppering refers to tiny, scattered brown dots seen on dermoscopy, often distributed homogeneously or focally in the superficial dermis or lesion, while the brown blotch or brown structureless areas observed in many tumors correspond to the increased epidermal pigmentation. Pseudonetwork and pigment network-like appearances were observed as melanin is interrupted by adnexal openings in the facial syringomas. Ovoid nests were observed in poroma, while no leaflike structures were noted in any adnexal tumor description [13]. 

Vascular patterns present a wide variety in adnexal tumors' dermoscopy. It is also important to note that skin pigmentation due to melanin can sometimes hide the visibility of blood vessels. Arborizing vessels, characteristic of BCCs, are frequently seen in trichoepitheliomas, cylindromas, and apocrine hydrocystoma, raising diagnostic challenges. Trichoepithelioma was the adnexal tumor mostly seen with arborizing vessels. Polymorphic vascular patterns, such as those seen in poromas, and dotted and looped vessels surrounded by a white halo were observed in eccrine poromas. Crown vessels remain the hallmark vessel pattern of sebaceous hyperplasia in patients with SoC [13]. Linear irregular vessels are more frequent in malignant or aggressive adnexal tumors, such as trichilemmal carcinoma and mucous adnexal carcinoma [14]. In the case of the latter one, the authors attribute this pattern to vessels growing in fibrous septa or capsules. Vessel distribution can be an indicator of differences between benign and malignant counterparts of the same adnexal entity. Sebaceous carcinoma has 'polymorphous vessels', which indicate that abundant blood vessels are located between the tumor nests and the epidermis and the tumor nests include sebum, while sebaceous adenoma usually manifests tortuous or linear telangiectasias at the periphery of the lesion, resembling more the location and type of the crown vessels of sebaceous hyperplasia. Vascular features like linear irregular vessels and hairpin vessels result from pressure due to the tumour mass and angiogenesis [3,19]. Therefore, adnexal tumors dermoscopy exhibits non-specific vascular features that clinicians cannot call for their identification; an observation that was made based on mixed populations. However, the presence of certain vascular patterns, such as polymorphic or irregular linear vessels, may indicate malignancy and needs to be co-evaluated with other features of the lesion.

Sebaceous tumors, such as sebaceous hyperplasia and sebaceous adenomas, predominantly display yellow structureless areas, due to their high sebum content. The presence of white or yellow structures often correlates with sebaceous and keratin-related differentiation. Sebaceous tumors frequently contain yellow-white globules or structureless areas, while trichoepitheliomas and pilomatricomas often exhibit homogeneous white areas and/or white streaks [9]. Milia-like cysts, frequently seen in trichoepitheliomas and syringomas, resemble those observed in seborrheic keratosis. The prominent milia-like white structures likely indicate follicular plugs, while the tiny dots, in the case of syringoma, represent eccrine ducts. In trichoepithelioma and trichoblastoma, these cysts may also correspond histopathologically to dermal keratocysts, that more readily visualized with a polarized dermoscope. Additionally, in the adnexal dermoscopy, the white, structureless area reflects the fibrous stroma, and the peripheral erythema corresponds to heightened vascularity [16].

The lesion's location is a crucial factor in differentiation. Poromas found on the palms and soles should not be mistaken for seborrheic keratosis or basal cell carcinoma (BCC), as these typically do not develop in such areas. Notably, pigmented poromas often appear on the lower leg, creating a diagnostic challenge when distinguishing them from pigmented BCC. Additionally, other skin cancer mimickers are rare in individuals with skin of color, an important consideration in diagnosis. For example, Bowen’s disease typically presents with brown lines and dots on dermoscopy, while keratoacanthomas exhibit a corneal plug and/or ulceration. Yellowish dermoscopic structures are primarily associated with sebaceous lesions such as sebaceous nevus, sebaceous carcinoma, sebaceous hyperplasia, sebaceous, sebaceous adenoma, and reticulated acanthoma with sebaceous differentiation, highlighting the utility of dermoscopy in assessment. Benign sebaceous tumors often resemble well-differentiated squamous cell carcinoma (SCC) or keratoacanthoma (KA).

Lastly, in cases of multiple lesions, conditions such as syringomas, milia, and xanthomas may mimic each other, but clinical and dermoscopic evaluation can help resolve these diagnostic challenges [3]. Differentiating adnexal tumors from nodular BCC in SoC poses a challenge due to overlapping features, particularly in vascular patterns. However, the distinct pearly appearance of BCC, which contrasts with the typically skin-colored presentation of adnexal tumors, along with the unique pigmentation features (with ovoid nests and blue, black, and grey dots being most common in BCC among patients with SoC) help guide the diagnosis [20].

## Conclusions

The dermoscopy findings of adnexal tumors, derived from studies of mixed populations, can also be present in dark-skinned individuals. It is worth noting that adnexal tumors did not exhibit a higher frequency of pigmented structures compared to those seen in fair skin tones, contrary to BCCs. The most common pigmentation trait found was brown blotches or brown structureless areas, corresponding to the increased epidermal pigmentation. In dark skin tones, the protective effect of melanin generally reduces the incidence of skin cancer, except in cases involving genetic mutations or systemic immunosuppression. Nevertheless, early detection and prompt diagnosis are crucial, particularly when distinguishing benign adnexal tumors from malignant skin cancers, as their differential diagnosis significantly impacts management and treatment outcomes.
